# Metrics for comparison of crystallographic maps

**DOI:** 10.1107/S1399004714016289

**Published:** 2014-09-27

**Authors:** Alexandre Urzhumtsev, Pavel V. Afonine, Vladimir Y. Lunin, Thomas C. Terwilliger, Paul D. Adams

**Affiliations:** a Centre for Integrative Biology, Department of Integrated Structural Biology, IGMBC, CNRS UMR 7104–INSERM U964–Université de Strasbourg, 1 Rue Laurent Fries, BP 10142, 67404 Illkirch, France; b Faculté des Sciences et Technologies, Université de Lorraine, 54506 Vandoeuvre-lès-Nancy, France; c Lawrence Berkeley National Laboratory, One Cyclotron Road, BLDG 64R0121, Berkeley, CA 94720, USA; d Institute of Mathematical Problems of Biology, Russian Academy of Sciences, Pushchino 142290, Russian Federation; e Los Alamos National Laboratory, Los Alamos, NM 87545-0001, USA; f Department of Bioengineering, University of California Berkeley, Berkeley, CA 94720, USA

**Keywords:** Fourier syntheses, crystallographic contour maps, map comparison, sigma scale, rank scaling, correlation coefficients

## Abstract

Numerical comparison of crystallographic contour maps is used extensively in structure solution and model refinement, analysis and validation. However, traditional metrics such as the map correlation coefficient (map CC, real-space CC or RSCC) sometimes contradict the results of visual assessment of the corresponding maps. This article explains such apparent contradictions and suggests new metrics and tools to compare crystallographic contour maps. The key to the new methods is rank scaling of the Fourier syntheses. The new metrics are complementary to the usual map CC and can be more helpful in map comparison, in particular when only some of their aspects, such as regions of high density, are of interest.

## Abstract 2

Rank scaling of Fourier syntheses leads to new tools for the comparison of crystallographic contour maps. The new metrics are in better agreement with a visual map analysis than the conventional map correlation coefficient.

## 1. Notation   

*F*(*hkl*)exp[*i*ϕ(*hkl*)]: crystallographic structure factor with indices *hkl*.

**F**_calc_ = *F*_calc_exp(*i*ϕ_calc_): structure factors calculated from an atomic model.

**F**_model_ = *F*_model_exp(*i*ϕ_model_): structure factors calculated from an atomic model including modelled contribution from bulk solvent and various scales (Afonine *et al.*, 2013 ▶).

*N* *_x_*, *N* *_y_*, *N* *_z_*: grid numbers defining a regular grid in real space.

*N*_grid_: total number of grid nodes of the unit cell used for comparison; in particular, *N*_grid_ = *N* *_x_* × *N* *_y_* × *N* *_z_* if the maps are analyzed for the whole unit cell.

**n** = (*n* *_x_*, *n* *_y_*, *n* *_z_*): grid node defined by its three integer indices.

ρ(*x*, *y*, *z*): Fourier synthesis calculated in the unit cell of direct space.

ρ(**n**) = ρ(*n* *_x_*, *n* *_y_*, *n* *_z_*): Fourier synthesis calculated in grid node **n**.

ρ_σ_(**n**) = ρ_σ_(*n* *_x_*, *n* *_y_*, *n* *_z_*): Fourier synthesis scaled in σ.

ρ_*d*1–*d*2_: Fourier synthesis calculated with structure factors in the resolution range (*d*_1_, *d*_2_).

ρ_complete_, ρ_incomplete_: Fourier syntheses calculated with a complete set of structure factors up to a given high-resolution cutoff or with some reflections excluded from this set; both the resolution value and the method used to exclude reflections are described explicitly for particular tests.

(*F*, ϕ) synthesis: Fourier synthesis calculated with the Fourier coefficients *F*exp(*i*ϕ).

*N*_μ_: number of grid nodes with the value below the cutoff level μ in the Fourier synthesis ρ: ρ(**n**) < μ; μ is given in the same units as ρ.

η(μ; ρ): quantile rank corresponding to the cutoff level μ for the Fourier synthesis ρ(**n**).

*Q*(**n**): (quantile) rank-scaled Fourier synthesis ρ(**n**).

*P*(**n**): rank-scaled Fourier synthesis ρ(**n**) with the values flattened out of the peaks.

*M*(*q*) = {**n**: *Q*(**n**) < *q*}: mask defined by the cutoff level expressed in the quantile rank *q*.

*D*(*q*; ρ_*a*_, ρ_*b*_); discrepancy function between two grid functions, ρ_*a*_(**n**) and ρ_*b*_(**n**), in particular between two Fourier syntheses.

CC(ρ_*a*_, ρ_*b*_): map correlation coefficient between two grid functions.

CC_r_(ρ_*a*_, ρ_*b*_): rank correlation coefficient between two grid functions.

CC_<*q*peak>_(ρ_*a*_, ρ_*b*_): peak correlation coefficient between two grid functions; selected peaks correspond to the *q*_peak_ quantile rank.

## 2. Introduction   

Macromolecular crystallography operates with the electron (or neutron) density distribution in crystals. For ideal crystals, this physical entity can be described by a periodic function ρ_exact_(*x*, *y*, *z*) of three space fractional coordinates (*x*, *y*, *z*) and can be represented by a Fourier series composed of an infinite number of complex coefficients *F*(*hkl*)exp[*i*ϕ(*hkl*)], 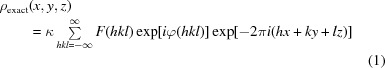
(Ewald, 1913 ▶). The values of these coefficients, called structure factors, depend on the crystal under study. The scale factor κ, equal to the inverse unit-cell volume, puts function (1) on an absolute scale; alternative scales can also be used. In crystallographic practice, Fourier series contain only a finite set *S* of terms and are usually calculated on a three-dimensional regular grid *N_x_* × *N_y_* × *N_z_* with the grid nodes described by integer indices **n** = (*n_x_*, *n_y_*, *n_z_*), 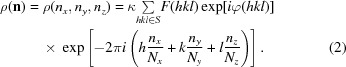
We call these grid functions (2) Fourier syntheses. To be analyzed visually or by a computer program, these mathematical entities are traditionally explored by contouring three-dimensional isosurfaces
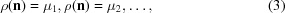
where μ_*n*_ are empirically chosen values. The result of such contouring is a geometric object that is referred to below as a crystallographic contour map.

Crystallographic structure solution typically deals with many maps arising at different stages of the process. Often, one is required to compare maps in order to assess model-building and/or refinement steps. Quantitative comparison of maps calculated for the same crystal, for different crystals and even for different structures is important to evaluate the progress of structure solution and to validate the structure. However, confusion about the three terms given above, electron (or neutron) density distribution, Fourier syntheses and corresponding Fourier contour maps, sometimes leads to apparent contradictions between numerical and visual analyses, as shown below.

As an example, we consider the exact electron density ρ_pept_*a*_(**n**) = ρ_exact_(**n**) corresponding to a peptide model (*B* = 1 Å^2^) placed in an orthogonal unit cell with unit-cell parameters *a* = *b* = 6, *c* = 3 Å, space group *P*1. ρ_pept_*b*_(**n**) is its Fourier synthesis at a resolution of 0.5 Å and ρ_pept_*c*_(**n**) is a Fourier synthesis calculated at a resolution of 1.0 Å for the same peptide model but taken with *B* = 5 Å^2^ and completed by a water molecule with *B* = 20 Å^2^.

**Figure 1:**
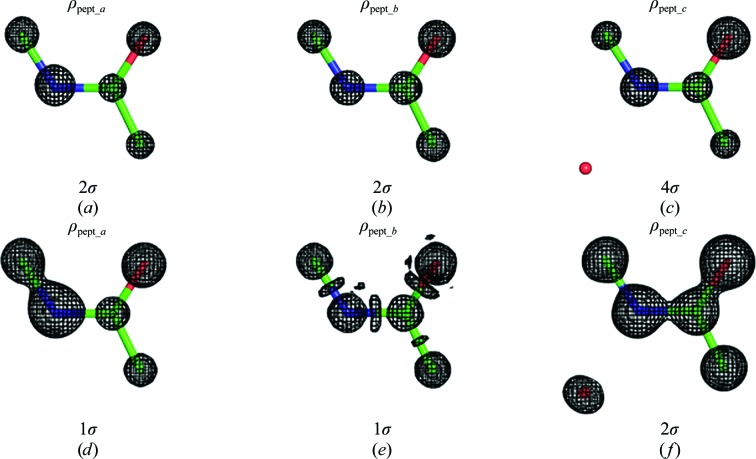
Fourier contour maps for artificial crystallographic peptide data. The function ρ_pept_*a*_(**n**) is an exact electron-density distribution for the peptide model with *B* = 1 Å^2^; ρ_pept_*b*_(**n**) is the corresponding Fourier synthesis at a resolution of 0.5 Å. ρ_pept_*c*_(**n**) is the Fourier synthesis at a resolution of 1.0 Å for the same model completed by a water molecule and taken with *B* = 5 Å^2^. All H atoms were excluded from the calculations.

The maps for ρ_pept_*a*_(**n**) and ρ_pept_*b*_(**n**) shown at 2σ (§[Sec sec3.1.1]3.1.1) are very similar to each other (compare Fig. 1 ▶ *a* with Fig. 1 ▶ *b*). However, the usual map correlation coefficient
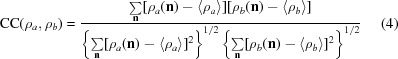
(see Supporting Information^1^ §S1) between ρ_*a*_(**n**) = ρ_pept_*a*_(**n**) and ρ_*b*_(**n**) = ρ_pept_*b*_(**n**) is only 0.90; here, 〈ρ*_a_*〉 and 〈ρ*_b_*〉 represent the mean values of ρ_*a*_(**n**) and ρ_*b*_(**n**), respectively. Indeed, the contour maps at 1σ (compare Fig. 1 ▶ *d* with Fig. 1 ▶ *e*) show that ρ_pept_*b*_(**n**) differs significantly from ρ_pept_*a*_(**n**). This reminds us that similarity of two contour maps at some cutoff level does not necessarily imply similarity of the corresponding syntheses.

Note that here we use the coefficient (4) to compare the whole syntheses, for example as in Read (1986 ▶) and Lunin & Woolfson (1993 ▶), while it can also be used locally (see, for example, Brändén & Jones, 1990 ▶; Kleywegt *et al.*, 2004 ▶; Rupp, 2006 ▶; Tickle, 2012 ▶).

Secondly, the traditional choice of a cutoff level in σ (§[Sec sec3.1.1]3.1.1) is often not appropriate for map comparison. The map for ρ_pept_*c*_(**n**) at 2σ (Fig. 1 ▶ *f*) shows a much larger volume of the unit cell in comparison with that for ρ_pept_*a*_(**n**) at the same 2σ cutoff level (Fig. 1 ▶ *a*). However, the maps look similar when taken at different cutoff values (compare Fig. 1 ▶ *c* with Fig. 1 ▶ *a*).

Thirdly, the three maps ρ_pept_*a*_(**n**), ρ_pept_*b*_(**n**) and ρ_pept_*c*_(**n**) look similar to each other, while the map correlation coefficient CC calculated using (4) is high for one pair of them, CC(ρ_pept_*a*_, ρ_pept_*b*_) = 0.9, and is low for another, CC(ρ_pept_*a*_, ρ_pept_*c*_) = 0.6.

In fact, the map correlation coefficient (4) is obtained by comparing two sets of values calculated on the same grid, comparing all these values point by point but with no reference to the position of these points in space (these may even be in a one-dimensional space). However, when we compare two maps visually we look at the shape of one or a few chosen isosurfaces. In other words, these two methods of comparison give different characteristics for different objects related to each other as explained above.

**Figure 2:**
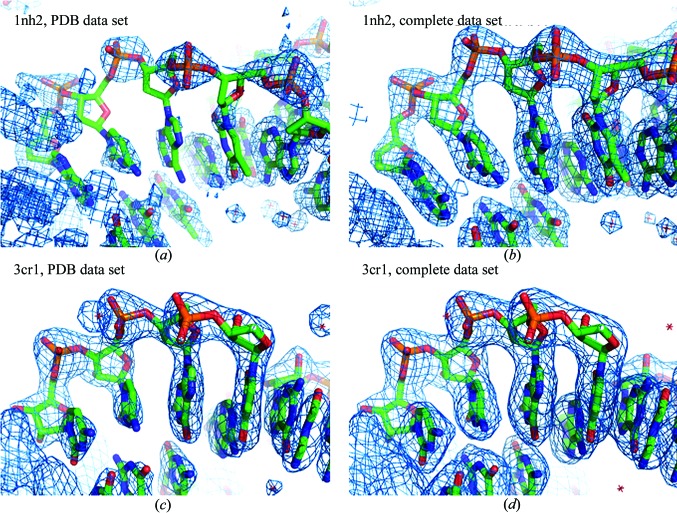
Fourier contour maps for 1nh2 and 3cr1. All syntheses are calculated with the model structure factors at a resolution of 1.90 Å (1nh2) or 2.25 Å (3cr1). The syntheses are obtained for the complete data sets (right column) and for those from the PDB (left column). The 1nh2 maps are shown with a cutoff level of 1.0σ and those for 3cr1 with a cutoff level of 1.5σ. See §[Sec sec4.2]4.2 for details. The map correlation coefficient between the top syntheses is 0.702 and that between the bottom syntheses is 0.642.

Fig. 2 ▶ illustrates a practical example with two protein models available in the PDB (Bernstein *et al.*, 1977 ▶; Berman *et al.*, 2000 ▶). Here again, the calculated CC values disagree with the visual analysis. The corresponding details are given in §[Sec sec4.2.1]4.2.1.

Crystallographers use contour maps at different contour levels to focus on different aspects of the maps. At high contour levels the most prominent features are shown, while at lower contour levels more details of the electron density are seen. In many cases it is the most prominent features that are most useful to the crystallographer in identifying where atoms are likely to be present in the structure. In other cases, of course, the details of the map are very important in identifying errors in atomic placement and in comparing different maps.

In this article, we focus on a subset of the information in a map, such as the prominent features in the electron density, and suggest new approaches to comparing crystallographic maps. The emphasis in this work is on the shapes of isosurfaces in these maps. These are the shapes that crystallographers normally use to identify the atomic features of structures in crystals.

Suppose we have two functions calculated on the same grid. For each function a mask can be defined by some isosurface, with all the points inside this mask having a value greater than the cutoff associated with the isosurface. We would like to compare the shapes of these masks (isosurfaces). Intuitively, masks containing a different number of grid nodes are different. The question we focus on is how similar are two masks composed of the same number of grid nodes, *i.e.* covering the same volume of the unit cell. We show below that to answer this question it is convenient to rescale the syntheses in the quantile rank (see §[Sec sec3.1.2]3.1.2) instead of a traditional scaling in σ (see §[Sec sec3.1.1]3.1.1).

After introducing rank scaling, we discuss a way to create a normalized metric useful in the comparison of two masks or a series of masks for various cutoff levels (§[Sec sec3.2]3.2). This naturally leads to a use of the Spearman rank correlation (Spearman, 1904 ▶; see also, for example, Lehmann & D’Abrera, 1998 ▶ and references therein), which is the same as the conventional correlation coefficient calculated for rank-scaled maps (§[Sec sec3.3]3.3). Considering only grid nodes with relatively high rank values results in another metric, a peak correlation coefficient (§[Sec sec3.4]3.4) that corresponds to a visual comparison of the contour maps and that is based on much of the key structural information in the maps. §[Sec sec4]4 gives various possible illustrations where the new metrics complement the traditional map correlation coefficient or explain some its apparent contradiction with a visual analysis.

Comparison of maps calculated on different grids is outside the scope of this work.

## 3. Methods   

### 3.1. Scaling of crystallographic Fourier syntheses   

#### 3.1.1. Scaling by σ   

In macromolecular crystallography, currently the most popular way of scaling crystallographic syntheses is by σ. Sigma-scaled Fourier syntheses are obtained as follows,
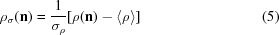
with
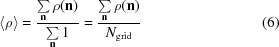
and
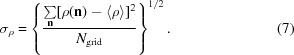
Here, ρ(**n**) is some initial function, *N*_grid_ is the number of grid points in the unit cell and 〈ρ〉 is always equal to 0 when the term *F*_000_ is absent from the Fourier series (2). With such a scaling, the grid function (5) has the properties
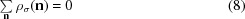
and
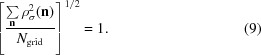
Empirically, crystallographers consider values of ρ_σ_(**n**) > 1 as a ‘signal level’ at which the structural details are analyzed (values notably above the mean value, *i.e.* above the value for bulk solvent) and values of ρ_σ_(**n**) > 3 as a ‘strong signal level’.

Another source of confusion comes from the map correlation coefficient (4). In statistics, the correlation coefficient is used to compare two sets of values from related distributions. However, the same formal expression is often used in crystallography, instead of the least-squares metric (Supporting Information §S1), to compare two syntheses defined as vectors in an *N*_grid_-dimensional space. We stress that in the current work we do not consider the crystallographic Fourier syntheses as random functions even when such a consideration has previously been used in a number of projects (see, for example, Luzzati, 1953 ▶; Blow & Crick, 1959 ▶; Ramachandran & Raman, 1959 ▶; Main, 1979 ▶ and references therein; Vijayan, 1980 ▶; Read, 1986 ▶; Lunin, 1989 ▶; Terwilliger, 2000 ▶; Burla *et al.*, 2010 ▶; Lang *et al.*, 2014 ▶). In the following, we consider that both the map correlation coefficient (4) and the new metrics are calculated for the whole unit cell. Naturally, they can be calculated locally for any part of the unit cell; in this case, *N*_grid_ would be the number of grid nodes inside this part.

Since the scaling (5)–(7) [Disp-formula fd6] [Disp-formula fd7] is a linear transformation, the correlation coefficient (4) calculated for the ρ_σ_(**n**) values coincides with the correlation coefficient CC calculated using the original values ρ(**n**).

While such scaling in σ is convenient to distinguish macromolecular features, it may be misleading when used for visual and numerical comparison of syntheses, as the example in §[Sec sec2]2 shows (Fig. 1 ▶; see also §[Sec sec4.1]4.1). The reason for this is that the frequency distribution of the values of the syntheses (Lunin, 1988 ▶, 1993 ▶; Main, 1990*a* ▶,*b* ▶) may be different for the two syntheses. As a consequence, the same cutoff level in σ defines different numbers of grid nodes selected by this level for these syntheses. Obviously, regions composed of a different number of points (using the same grid) can never be equal.

#### 3.1.2. Rank scaling   

The map comparison becomes easier if the Fourier syntheses are scaled in quantile ranks or are rank scaled. In image processing, this operation is referred to as histogram equalization (see, for example, Pratt, 1978 ▶). This means that for each cutoff value μ we count the number *N_μ_* of grid nodes **n** such that the synthesis value is below it, ρ(**n**) < μ, and we then calculate the ratio
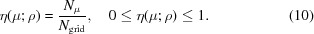



Here, the second argument, ρ, is the Fourier synthesis to be studied and the first argument, μ, is a particular value. In statistics, the value η (10) is called a quantile rank; when multiplied by 100 this gives the percentile rank. The notions of percentile and quantile and the corresponding ranks have recently been used in crystallography by Pozharski (2010 ▶), Gore *et al.* (2012 ▶) and Tickle (2012 ▶), although for different goals. Previously in crystallography, a scaling in units complementary to the quantile/percentile rank, *i.e.* in the fractional unit-cell volume covered by the mask ρ(**n**) > μ, has been used by Vagin (personal communication) and by Lunin and coworkers (Lunin, 1988 ▶; Vernoslova & Lunin, 1993 ▶).

For a given synthesis ρ, the function (10) increases with μ. This monotonic behaviour permits an easy rank scaling (Appendix *A*), replacing the value ρ(**n**) at each point by
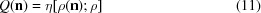
using η(μ; ρ) (10). This scaling does not change the shape of any isosurface, as all points with the same value of μ have the same value of the new function. Note that in contrast to the rescaling in σ, rank rescaling is a nonlinear transformation.

**Figure 3:**
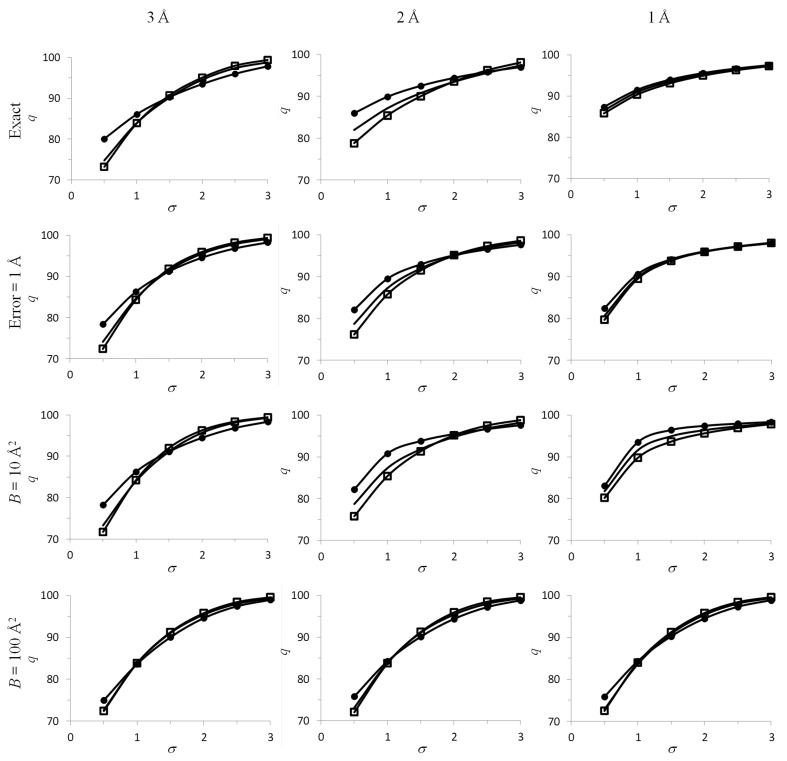
Quantile ranks (multiplied by 100) corresponding to a different σ cutoff in Fourier syntheses at resolutions of 3 Å (left column), 2 Å (central column) and 1 Å (right column). Syntheses are computed with the exact structure factors calculated from an accurate atomic model (top row) and from a model with large random coordinate errors (second row) with a small (third row) and a large (bottom row) atomic displacement parameter (*B*). The model was placed in unit cells of different sizes simulating different percentages of bulk-solvent content equal to 0.23 (empty square markers), 0.58 (no markers) and 0.81 (solid circle markers).

Most commonly, macromolecular crystallographers work with syntheses calculated with the coefficients (amplitudes) *wF*_obs_ or 2*mF*_obs_ − *DF*_calc_ (Read, 1986 ▶) and scaled in σ. Analyzing these syntheses, at least for the resolutions 1–3 Å at which many structural projects are carried out, the cutoff values μ used for visual interpretation range approximately between 1 and 2σ. The particular choice may depend on the resolution, bulk-solvent content and other factors. Fig. 3 ▶ shows that the ranks corresponding to these values vary approximately from 0.85 to 0.95. These model calculations agree with calculations using various experimental data (not shown); in particular, this includes experimental data from PDB entries with low, medium and extremely high solvent content. This also agrees with the previous observation by Ioerger & Sacchettini (2002 ▶).

Other scaling methods, *e.g.* choosing another κ value [for example such that max_**n**_|ρ(**n**)| = 100 or using a so-called ‘absolute scale’] or another nonlinear scheme (for example, Bhat, 1988 ▶; Lunin *et al.*, 2000 ▶) are known, but we will not review this issue here in detail.

### 3.2. Comparison of two masks   

Since the introduction of graphics stations in macromolecular crystallography, syntheses have typically been presented by a single isosurface at a time with the possibility of varying the corresponding cutoff levels. When we compare two syntheses visually, we look at the shape of the masks covered by the corresponding isosurfaces (there are a number of publications on image analysis that discuss the relevant computational procedures; see, for example, Bruckner & Möller, 2010 ▶ and references therein). As mentioned above, the quantitative similarity of two masks can be examined most readily when these masks are constructed so that they contain equal volumes. This is a particular advantage of the rank-scaling approach, which naturally leads to equal volumes at given contour levels in different maps. Other one-to-one syntheses-scaling schemes with a similar property (Supporting Information §S3) are less convenient for the goals of the current work.

In order to compare two masks, we start by measuring (calculating) the difference between them. Let *Q_a_*(**n**) and *Q_b_*(**n**) be the two rank-rescaled syntheses ρ*_a_*(**n**) and ρ*_b_*(**n**). For any quantile rank value, 0 ≤ *q* ≤ 1, the subsets (masks)
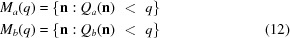
contain the same number *N*_selected_ = *qN*_grid_ of grid nodes. The difference between these masks may be described by the number *N*_diff_ of the nodes that belong to one of them and do not belong to another one, 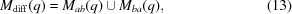
where
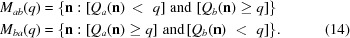
Note that by construction *M_ab_*(*q*) and *M_ba_*(*q*) contain the same number of points. The condition *N*_diff_ = 0 means that the masks *M_a_*(*q*) and *M_b_*(*q*) coincide. If *N*_diff_ > 0 then the masks are different, but the value of *N*_diff_ does not allow judgment of the degree of this difference because the same number of differing points *N*_diff_ may have a different significance for small and large rank values *q*.

To put this difference *N*_diff_ on a scale, we compare *M_a_*(*q*) with a random set *M*_random_ composed of the same number *N*_selected_ of grid nodes distributed uniformly in the unit cell and thus containing no structural information. On average, the number of grid nodes of *M*_random_ that are outside *M_a_*(*q*) is just the number of grid nodes in *M*_random_ multiplied by the fraction of the cell that is outside *M_a_*(*q*), *i.e.* by (1 − *q*),
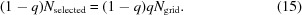
The same estimate is valid for the comparison of *M*_random_ with *M_b_*(*q*). Based on this, we normalize *N*_diff_ as
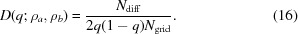
The calculated values of the normalized function *D*(*q*; ρ_*a*_, ρ_*b*_) at some value of the argument *q* may be equal to its minimal possible value, zero, when the corresponding masks coincide and may approach one when the two masks are uncorrelated. Since (15) is only a statistical estimate, in practice *D*(*q*; ρ_*a*_, ρ_*b*_) may sometimes happen to be greater than one. We notate (16) as *D*(*q*; ρ_*a*_, ρ_*b*_) and not *D*(*q*; *Q*_*a*_, *Q*_*b*_) to stress that this measure can be applied to any two functions calculated on the same grid and not necessarily functions rescaled in some specific way. We call (16) a discrepancy function. Different values of the argument *q* are useful for obtaining different types of information: high *q* values are useful for identifying the peaks of the functions (atomic positions or macromolecular chain), while *q* close to 0.5 is useful for the identification of molecular envelopes (the actual corresponding value of *q* varies with the fraction of the solvent region).

### 3.3. Rank correlation coefficient   

When calculating the discrepancy function *D* (16) between two syntheses, we compare masks of equal size (‘equivalent masks’), varying the cutoff level at which these masks are selected. To make such comparison easier, we rank-scale the syntheses. When comparing a pair of equivalent masks we check each grid node one by one, identifying whether this grid node is inside only one mask, the other, both or neither.

Alternatively, after rank scaling the two syntheses *Q* *_a_*(**n**) and *Q* *_b_*(**n**) we may express their similarity by
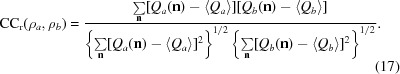
This metric of similarity of syntheses ρ*_a_*(**n**) and ρ*_b_*(**n**) varies from −1 to 1, and in statistics it is known as Spearman’s rank correlation coefficient (Spearman, 1904 ▶). We may note that (Appendix *A* [App appa])
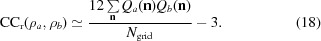
The key property of the rank correlation coefficient CC_r_(ρ_*a*_, ρ_*b*_) is its invariance with respect to scaling of the syntheses ρ*_a_*(**n**) and ρ*_b_*(**n**). As mentioned in §[Sec sec3.1.1]3.1.1, scaling by σ does not change the standard correlation coefficient CC(ρ_*a*_, ρ_*b*_). In particular, CC(ρ_*a*_, ρ_*b*_) = 1 for all proportional functions, *i.e.* when ρ*_b_*(**n**) = λρ*_a_*(**n**) for all **n**. An important advantage of the rank correlation coefficient CC_r_(ρ_*a*_, ρ_*b*_) compared with CC(ρ_*a*_, ρ_*b*_) is that the former is invariant upon any monotonic (and not necessary linear) rescaling of the syntheses ρ*_a_*(**n**) and ρ*_b_*(**n**). In particular, CC_r_(ρ_*a*_, ρ_*b*_) for a pair of nonproportional functions related by any monotonously increasing function ρ*_b_*(**n**) = *f*[ρ*_a_*(**n**)].

Note that using CC_r_(ρ_*a*_, ρ_*b*_), in contrast to *D*(*q*; ρ_*a*_, ρ_*b*_), applies not only to Fourier maps shown as series of masks but also to any continuous spectrum of colours or intensities (see, for example, Schotte *et al.*, 2003 ▶).

As an example, the rank correlation coefficients CC_r_ for the peptide syntheses defined in §[Sec sec2]2 are given by CC_r_(ρ_pept_*a*_, ρ_pept_*b*_) = 0.56 and CC_r_(ρ_pept_*a*_, ρ_pept_*b*_) = 0.22, which is more indicative of their difference than the standard map correlation coefficient values, which are equal to 0.90 and 0.60, respectively. More details of comparison of these syntheses using the discrepancy function, the rank correlation coefficient and other metrics as defined below are discussed in §[Sec sec4.1]4.1.

### 3.4. Comparison of peaks   

Syntheses such as *wF*_obs_ or 2*mF*_obs_ − *DF*_calc_ scaled in σ have both positive and negative values. While analysis of negative values may be important (see, for example, Urzhumtsev *et al.*, 1989 ▶), often only the regions of positive values are of interest. This is the case for visual analysis and manual model building; for example, the program *Coot* (Emsley *et al.*, 2010 ▶) defaults to showing nondifference σ-scaled maps at μ > 0. However, maps similar in the positive domain may be different in the negative domain. This may give rise to an apparent contradiction: similar-looking maps (inspected in the positive domain only) may have low correlations computed using the entirety of the maps.

Since map regions with high values contain most of the structural information, it is useful to have a way to compare contour maps such that (i) differences between low values of the synthesis should not play a role and (ii) if a high value in one synthesis corresponds to a low value in another synthesis, the desired metric should not depend on the exact value of the lower value.

For example, for structures with the most frequent percentage of bulk solvent, the separation of positive and negative values in σ-scaled maps roughly corresponds to half of the syntheses, *i.e.* to the quantile-rank cutoff *q* = 0.50. When comparing the top halves of the rank-scaled syntheses *Q_a_*(**n**) and *Q_b_*(**n**), we shall exclude from comparison all grid points for which the values in both syntheses are low, defining a set of grid nodes staying with
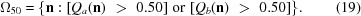
Similarly, to effectively compare regions with high density (near peaks in the map) corresponding to a higher quantile rank value 0.5 < *q*_peak_ < 1.0, we define
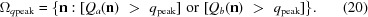
We then flatten the syntheses values in the Ω_*q*peak_ points if these values are below *q*_peak_ for one of the syntheses,
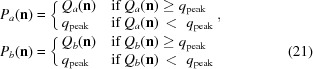
and finally calculate 
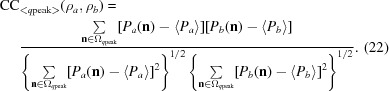
Here,
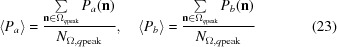
and *N*_Ω,*q*peak_ is the number of grid nodes in Ω_*q*peak_ defined by (20). For example, a *q*_peak_ value equal to 0.50 defines CC_50_ and a *q*_peak_ value equal to 0.90 defines CC_90_ . As previously, the sums in (22) exclude all grid nodes in which both syntheses have values lower than the chosen threshold, indicating that we are not interested in comparison of syntheses at these points.

### 3.5. Practical applications   

Depending on the particular problem, different tools are useful to compare crystallographic Fourier syntheses and the corresponding contour maps.

Naturally, when the similarity of three-dimensional functions (for example, crystallographic Fourier syntheses) is analyzed, for example when these functions are used to extract the phase values of corresponding Fourier coefficients, the traditional map correlation coefficient (4) is still a good metric.

However, in a major part of crystallographic projects only the Fourier contour maps for positive cutoff values (in σ-scaled syntheses) are used for visual inspection of maps. Moreover, for syntheses at a resolution of 1–3 Å the most frequently used cutoff levels of 1–2σ correspond to rank values *q* of as high as 0.85–0.95. To accompany the traditional visual analysis, we suggest using the coefficient CC_90_ as a rule of thumb and switching to CC_95_ using higher rank values in the case of a larger fraction of bulk solvent, higher map resolution or smaller *B* factors, and switching to CC_85_ or CC_80_ in the opposite situations. The correlation coefficient CC_50_ may be used to characterize the similarity of isosurfaces roughly corresponding to molecular masks for structures with typical values of the bulk-solvent fraction.

Use of the coefficient CC_r_ may be advised when the whole set of isosurfaces, including those for negative peaks, are studied. The discrepancy function *D*(*q*; ρ_1_, ρ_2_) completes this toolset when more detailed information is required.

§[Sec sec4]4 below provides examples of applications of the new correlation coefficients to macromolecular diffraction data. All of these applications confirm that the new metrics reflect important synthesis details that the standard CC does not fully consider. Moreover, in some cases they explain an apparent disagreement between CC and visual map analysis.

With regard to an appropriate visual comparison of syntheses, we suggest rank-scaling them first and selecting the same cutoff value for the visualization of each. Alternatively, the syntheses can be taken on their initial scales (for example in σ) with the cutoff levels selected from equalization of the corresponding rank values as described in (28) and (29) in Appendix *A* [App appa].

## 4. Examples, applications and results   

### 4.1. Peptide model data   

We first apply the new metrics to the syntheses ρ_pept_*a*_(**n**), ρ_pept_*b*_(**n**) and ρ_pept_*c*_(**n**) defined in §[Sec sec2]2 for a simulated peptide crystal. For the very sharp electron-density distribution ρ_pept_*a*_(**n**) corresponding to a crystal with very few atoms, the rank scale is lower than that for the macromolecular syntheses at usual resolutions of 1–3 Å. In particular, for ρ_pept_*a*_(**n**) the value *q* = 0.80 corresponds to a zero cutoff level in σ, the value *q* = 0.95 corresponds to 0.6σ and *q* = 0.99 corresponds to 2.2σ. (Fig. 3 ▶ reminds us that for typical macromolecular syntheses the value 0σ corresponds to the range *q* = 0.40–0.60, the value 1σ corresponds to the range *q* = 0.85–0.90 and 2σ to the range *q* = 0.90–0.95.)

For the exact electron-density distribution ρ_pept_*a*_(**n**) and the corresponding synthesis ρ_pept_*b*_(**n**) at a resolution of 0.5 Å, the rank correlation coefficient is lower than the standard map correlation coefficient (Table 1 ▶). This means that for most cutoff levels the masks in the 0.5 Å resolution synthesis differ significantly from those in the exact electron density. Figs. 1 ▶(*d*) and 1 ▶(*e*) provide an example. The coefficient CC_90_(ρ_pept_*a*_, ρ_pept_*b*_) is above 0.80, indicating that the peaks (their position and shape) around atomic positions are more or less conserved.

Both the CC and CC_r_ correlation coefficients for ρ_pept_*a*_(**n**) with ρ_pept_*c*_(**n**) are lower than for the comparison of ρ_pept_*a*_(**n**) with ρ_pept_*b*_(**n**); this is owing to the lower resolution of ρ_pept_*c*_(**n**) and the presence of an additional atom in the crystal. Neither of these values indicates similarity of the contour maps showing peaks above the level for the water molecule (Figs. 1 ▶ *a* and 1 ▶ *c*), while the correlation coefficient CC_95_ does.

The Supporting Information (§S2) contains another example built on the basis of this peptide model; this example is more mathematical and illustrates comparison of grid functions by different correlation coefficients in a more transparent way. These results confirm that the new metrics describe the information contained in the crystallographic contour maps much better than the traditional metrics.

### 4.2. Incomplete low-resolution data sets   

#### 4.2.1. Explaining an apparent contradiction between low correlation coefficients and similar contour maps   

A model *F*_calc_exp(*i*ϕ_calc_) Fourier synthesis (referred to as ρ_incomplete_) computed for PDB entry 1nh2 by (2) using reflection indices from the deposited data set (Bleichenbacher *et al.*, 2003 ▶; highest resolution 1.9 Å; data completeness 95%) shows part of the structure very poorly (Fig. 2 ▶ *a*). Fig. 2 ▶(*b*) shows a model Fourier synthesis ρ_complete_ calculated with the same coefficients using all theoretically possible reflections up to 1.9 Å resolution. The correlation coefficient CC calculated using (4) between the two syntheses is 0.70. Since both syntheses were calculated with the model data, the only source of difference is the missing reflections, essentially the lowest resolution reflections (there are 300 reflections missing from 408 with resolution below 10 Å; all 59 reflections with resolution below 20 Å are missing).

A similar comparison of ρ_incomplete_ with ρ_complete_ for another test case (PDB entry 3cr1; MacElrevey *et al.*, 2008 ▶; highest resolution 2.25 Å; data completeness 98%) yields an even lower map correlation coefficient CC = 0.64, which one would expect to be reflected by a larger difference between the two maps. This low correlation coefficient is owing to missing only 2% of the reflections (there are 116 reflections missed out of 251 collected at a resolution below 10 Å and 32 reflections out of 42 at a resolution below 20 Å). However, the contour map obtained with the incomplete data set is perfectly interpretable for the whole molecule and is very similar to the map calculated with the complete set of reflections (compare Fig. 2 ▶ *d* with Fig. 2 ▶ *c*). This illustrates that the map correlation coefficient is not necessarily a good predictor of the visual similarity of maps either from its value or when comparing different pairs of maps.

The rank correlation coefficient CC_r_ (18) is 0.30 for 1nh2 and just 0.01 for 3cr1 and is even lower than the values of the standard map correlation coefficient. It shows that in this case of missing low-resolution data most of the masks are severely changed compared with the corresponding masks in ρ_complete_ (see also Urzhumtsev, 1991 ▶; Urzhumtseva & Urzhumtsev, 2011 ▶).

**Figure 4:**
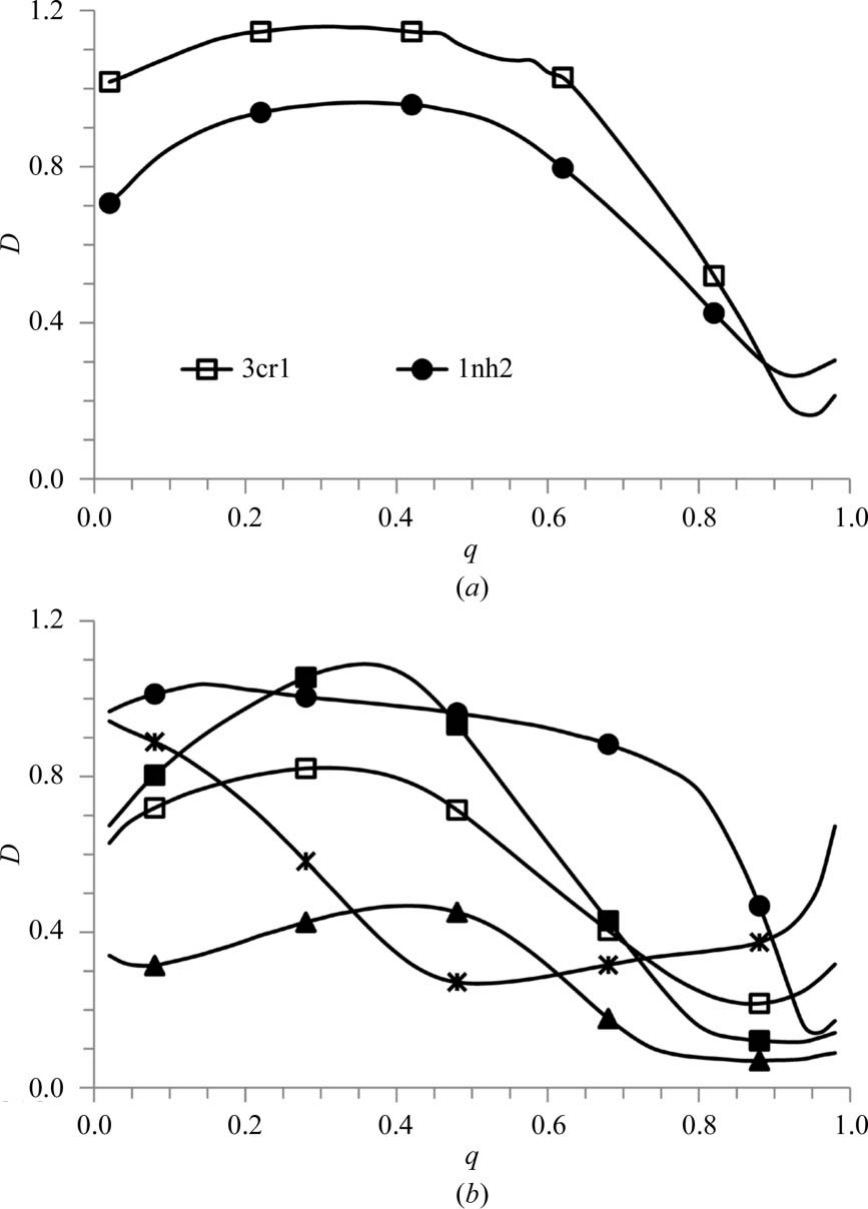
Discrepancy function *D*(*q*) comparing the Fourier contour maps obtained with complete and incomplete data sets. (*a*) Comparison of the syntheses ρ_incomplete_ calculated for the set of reflections as deposited in the PDB with the syntheses ρ_complete_ obtained with the complete data set of the respective resolution (*d*_high_ = 1.90 Å for 1nh2 and *d*_high_ = 2.25 Å for 3cr1). (*b*) Comparison of ρ_complete_ (complete data set at a resolution from *d*_high_ to infinity) with ρ_incomplete_ calculated with a data set in the resolution interval *d*_high_ to 10 Å. The curves are shown for *d*_high_ = 2 Å (solid squares, 1zud; solid circles, 1q09; solid triangles, 1ous) and for *d*_high_ = 4 Å (open squares, 1zud). The curve marked by stars is for comparison of the two ρ_complete_ maps for 1zud, one calculated with the complete data set at a resolution of 2 Å and the other with the complete data set at a resolution of 4 Å.

The peak correlation coefficient considers only the part of the map in the quantile rank greater than 0.90 and gives different information. Its value is 0.67 for 1nh2 and 0.83 for 3cr1 and shows that the peaks are conserved much better for 3cr1, agreeing with the visual analysis. This relationship is not shown by either the standard map correlation coefficient or the rank correlation coefficient. Fig. 4 ▶(*a*) expands on this calculation of CC_90_ by showing the discrepancy function *D*(*q*) for these two comparisons. It can be seen that for most rank values *q* the contours are quite different in both cases, *D*(*q*) ≃ 1, that for high *q* values such as 0.90 they are equally similar and for very high values such as *q* ≃ 0.95 they are more similar for 3cr1.

#### 4.2.2. Effect of low-resolution incompleteness on crystals with various solvent contents   

The examples in §[Sec sec4.2.1]4.2.1 illustrate the effect of low-resolution data incompleteness. It seemed possible that the strength of this effect might depend on the fraction of bulk solvent in the crystal. We made a comparative analysis considering three cases of bulk-solvent content: near the very common value of 50% (PDB entry 1zud; Lehmann *et al.*, 2006 ▶; solvent content 0.47), very high (PDB entry 1q09; Changela *et al.*, 2003 ▶; solvent content 0.84) and very low (PDB entry 1ous; Loris *et al.*, 2003 ▶; solvent content 0.24).

For each of these structures, we calculated a complete set of structure factors *F*_calc_exp(*i*ϕ_calc_) from the atomic model at a resolution of 2 Å. We call the Fourier synthesis calculated with these structure factors ρ_2–∞_(**n**) = ρ_complete_(**n**). We also calculated another Fourier synthesis *F*_calc_exp(*i*ϕ_calc_) in which all of the structure factors at a resolution outside the range 2–10 Å were excluded. We call this synthesis omitting low-resolution data beyond 10 Å ρ_2–10_(**n**) = ρ_incomplete_(**n**).

Both the conventional correlation coefficient CC and the rank correlation coefficient CC_r_ comparing ρ_2–∞_(**n**) and ρ_2–10_(**n**) decrease with increasing volume of the bulk-solvent region in these cases (Table 2 ▶). Note that for 1ous, with an extremely low bulk-solvent content, all of the maps are well conserved.

**Figure 5:**
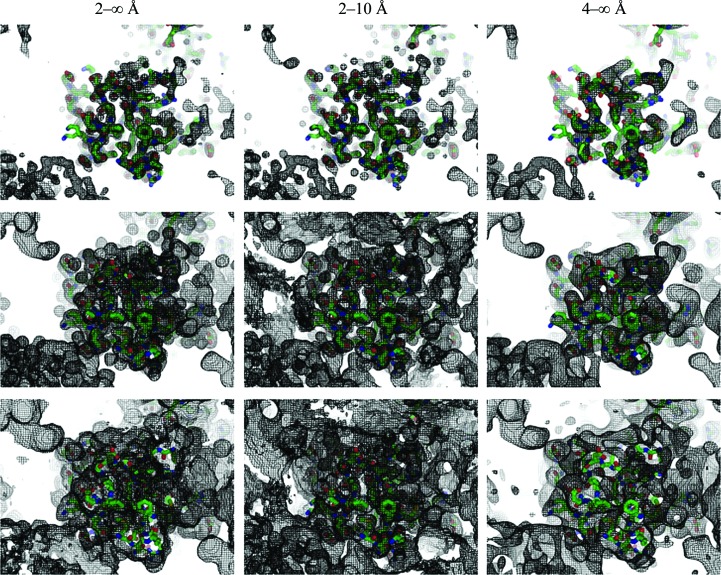
Fourier contour maps for 1zud. All syntheses are calculated with the model structure factors at the resolution cutoff indicated above each column and are shown at different rank levels (top, 0.9; middle, 0.7; bottom, 0.5). The solvent content is 0.47. Note the conservation of peaks and the loss of the molecular envelope when removing low-resolution data and the conservation of the envelope with decreasing resolution.

The variation in CC_r_ is more significant; in particular, its value of close to zero for 1q09 means that for these data most of the masks changed, information which is difficult to extract from the CC value of above 0.7. At the same time, the peaks are well conserved for all three structures; Fig. 5 ▶ gives an example for 1zud. The larger the bulk-solvent content, the higher the quantile rank corresponding to the highest value of the peak correlation coefficient (Table 2 ▶; Fig. 4 ▶ *b*).

The situation is quantitatively similar when we compare the corresponding maps ρ_4–∞_(**n**) = ρ_complete_(**n**) and ρ_4–10_(**n**) = ρ_incomplete_(**n**) calculated with data at lower resolution, in the ranges from 4 Å to infinity and from 4 to 10 Å, respectively (Table 2 ▶).

### 4.3. Effect of data-resolution cutoff   

Intuitively, it is clear that excluding high-resolution data changes the maps in a different way than excluding low-resolution data. It is easy to illustrate this using the new metrics.

To do so, for each of the three structures described in §[Sec sec4.2]4.2 we calculated the *F*_calc_exp(*i*ϕ_calc_) syntheses ρ_2–∞_(**n**) and _4–∞_(**n**) with the complete data sets at resolutions of 2 and 4 Å, respectively, and compared them. The map correlation coefficient values CC(ρ_2–∞_, ρ_4–∞_) are relatively high; for example for 1q09 this coefficient is as high as 0.88. This number shows some difference in the maps at such high- and low-resolution cutoffs; however, one might intuitively expect a much larger difference. Indeed, the rank correlation coefficient CC_r_(ρ_2–∞_, ρ_4–∞_) is much lower for 1q09, being equal to 0.44 and showing that the maps are substantially different.

As expected, the peak correlation coefficients for high rank values *q* are low (see, for example, CC_95_ and CC_99_) since the peaks are merged in the 4 Å resolution maps in comparison with the 2 Å resolution maps. The close-to-zero values of these coefficients are more intuitive than the value of the map correlation coefficient CC(ρ_2–∞_, ρ_4–∞_) given above.

At the same time, some peak correlation coefficients are relatively high, *e.g.* CC_80_(ρ_2–∞_, ρ_4–∞_) = 0.85 for 1q09. The corresponding rank value corresponds well to that defining the molecular region (see also Fig. 5 ▶) and shows that the molecular masks are less affected by excluding the high-resolution data. For the 1ous data, the molecule occupies practically the whole unit cell (and simply the whole unit cell if structural waters are included), and all peak correlation coefficients for it are low, showing changes in the maps at all cutoff levels.

Thus, using CC_r_ and the rank correlation coefficients may illustrate features that are difficult to see when referring only to the standard map correlation coefficient CC (4).

### 4.4. Effect of excluding reflections for cross-validation   

§[Sec sec4.2]4.2 shows that the loss of a relatively small number of low-resolution reflections (as few as 2%) can result in significant changes in the Fourier contour maps. On the other hand, the test data set (Brünger, 1992 ▶), typically containing 5–10% of the total number of reflections, is purposely excluded from all calculations; this should be the case for all steps including, formally speaking, the calculation of contour maps (although the latter is not always the case in practice). These data are used for validation (Brünger, 1992 ▶) and to estimate statistical parameters (Lunin & Skovoroda, 1995 ▶; Pannu & Read, 1996 ▶; Murshudov *et al.*, 1997 ▶). In general, the reflections for the test set are chosen randomly and uniformly across reciprocal space.

There is an old and frequently asked question whether excluding such reflections noticeably distorts the Fourier contour maps. We do not analyze this question in detail here, but simply illustrate the effects for a typical protein structure under typical conditions. To do so, we used the IF2 structure that we recently solved (Simonetti *et al.*, 2013 ▶; PDB entry 4b3x). The corresponding crystals belonged to space group *P*2_1_2_1_2_1_, with unit-cell parameters *a* = 45.42, *b* = 61.46, *c* = 162.40 Å. The experimental data set is complete to 2 Å resolution (with only two low-resolution reflections missing); bulk solvent occupies approximately 50% of the unit cell. The *R* and *R*_free_ values calculated by *PHENIX* (Adams *et al.*, 2010 ▶) are less than 0.18 and 0.22, respectively, showing that the structure factors *F*_model_exp(*i*ϕ_model_) calculated from the atomic model including the correction from the bulk solvent [Jiang & Brünger (1994) ▶, with the improvements described by Afonine *et al.* (2013 ▶)], reproduce the experimental data well. Thus, we used the phase values ϕ_model_ as the best possible approximation to the unknown values to be associated with the experimental structure-factor amplitudes *F*_obs_.

We calculated a series of Fourier syntheses at a resolution of 2 Å with coefficients *F*_obs_exp(*i*ϕ_model_), with the fraction of randomly excluded reflection ranging between 5 and 10%, as is routinely undertaken for test-set reflections. Each of these syntheses was compared with a synthesis calculated with the complete data set. The correlation coefficient CC between them remained high, *i.e.* above 0.90, even when the test set contained up to 20% of the data. However, the peak correlation coefficients CC_50_–CC_80_ indicated non-negligible map changes when 10% of the data were excluded. The maps showed significant noise at the rank value *q* = 0.80 (roughly 0.4σ for this synthesis) and incorrect density for a few weakly defined side chains. We note that the molecule occupies approximately half of the unit cell: *q* = 0.50. The differences resulting from the exclusion of 10% of reflections are more significant than the differences owing to experimental errors in amplitudes, as can be seen from comparison with the maps calculated with coefficients *F*_model_exp(*i*ϕ_model_) (Table 3 ▶). Overall, maps obtained with the model data *F*_model_exp(*i*ϕ_model_) illustrated a behaviour similar to that for *F*_obs_exp(*i*ϕ_model_) maps.

Summarizing, we suggest that carrying out an analysis of the rank and peak correlation coefficients could be used as a routine tool for identifying a suitable fraction of reflections for a test set in Fourier syntheses even when this set has been already assigned. A synthesis may be calculated with the working set of reflections and with the full available data set, and if the rank or peak correlation coefficients between these maps are low (a more systematic analysis is probably required to define appropriate critical values), the test data set might be reduced for further calculations by reassigning, also randomly and uniformly, some reflections back to the working set. As this example shows, the usual correlation coefficient alone may be not sufficiently informative.

### 4.5. Bulk-solvent contribution   

It is largely accepted that using a bulk-solvent correction is vital in order to properly include low-resolution data into the structure-solution process (see, for example, Phillips, 1980 ▶; Fenn *et al.*, 2010 ▶; Afonine *et al.*, 2013 ▶ and references therein). However, the influence of the bulk-solvent correction on Fourier syntheses has been less discussed.

To analyze the direct effect of the bulk-solvent contribution on the Fourier synthesis, complementary to the synthesis with {*F*_model_exp(*i*ϕ_model_)} for the IF2 model (§[Sec sec4.4]4.4), we calculated another synthesis with the structure factors {*F*_calc_exp(*i*ϕ_calc_)} without a bulk-solvent correction. The data sets were complete at the resolution of 2 Å. As mentioned above, the first data set, including the bulk solvent, reproduces the experimental data quite well.

The correlation coefficient CC between the two syntheses, equal to 0.89, indicates their high similarity. However, the rank coefficient CC_r_ of 0.62 shows that in fact the changes in the map owing to unmodelled bulk solvent are not negligible. This means that ignoring a bulk-solvent correction when modelling the ‘experimental syntheses’ may result in maps that differ from the correct maps and therefore may lead to wrong or unjustified conclusions. In particular, such data are not recommended for analysis of molecular envelopes since they may be mostly affected by this improper modelling (Table 4 ▶). At the same time, such simulated syntheses can be successfully used when studying only the structural details since CC_80_–CC_95_ indicate very high similarity of the peaks.

Comparison of the corresponding syntheses calculated at a resolution of 3 Å gives values comparable with those for the 2 Å resolution syntheses. However, the peak correlation coefficients for the rank *q* ≥ 0.9 are lower. For example, the coefficient CC_99_ corresponding roughly to the 3σ cutoff level decreases from 0.95 at 2 Å to 0.86 at 3 Å. This indicates that at lower resolution limits the unmodelled bulk-solvent contribution may distort not only the molecular envelopes but also the peaks of the syntheses.

## 5. Discussion   

The several examples presented in this work show that the traditional map correlation coefficient CC does not always correspond well to the similarity of or the difference in two Fourier syntheses based on visual examination. Approaches are presented to address this problem. They are based on the concept of a rank scaling of the syntheses. With such a scaling, regions selected with the same cutoff level contain the same number of grid nodes and the number of grid nodes in common is a useful measure of the similarity of the maps at that cutoff level.

The rank correlation coefficient CC_r_ is calculated as a correlation of the rank-scaled syntheses instead of the initial values ρ(**n**), for example those in σ. Both CC and CC_r_ are equal to 1 when the values of the two syntheses are related by a linear transformation. However, in contrast to CC, CC_r_ is equal to 1 also when the values of the syntheses are related by a nonlinear monotonic transformation; here, the maps are exactly the same but correspond to different cutoff levels on the original scales.

To accompany traditional visual analysis, we suggest using the peak correlation coefficients, in particular CC_90_, as a rule of thumb, adjusting the peak level to particular situations and problems. To compare molecular masks or peaks in the low-resolution maps, the correlation coefficient CC_50_ may be more appropriate. The discrepancy function *D*(*q*; ρ*_a_*, ρ*_b_*) compares the selected regions (masks) by counting the number of grid nodes in complementary regions, regardless of the exact values of the syntheses in these nodes.

The computational tools described here may be applied to answer additional questions to those that we have illustrated. The new coefficients may be calculated not in the whole unit cell but locally in a given region. With the peak correlation coefficient, one may compare syntheses previously difficult to compare numerically such as the usual σ_A_ synthesis and a difference synthesis. These tools may be used, in the case of comparing several maps, to select the one for which the corresponding contour maps correspond better to a control map. Naturally, the choice of the map for comparison is important and should be considered for each particular project.

The developed metrics can be also applied to compare maps corresponding to different crystals or to noncrystallographic objects, for example electron microscopy reconstructed images. The only requirement is that the compared parts of the images are of the same size and the maps are calculated on the same grid.

The tools discussed in this manuscript, namely the discrepancy function *D*(*q*; ρ*_a_*, ρ*_b_*), the rank correlation coefficient CC_r_(ρ*_a_*, ρ*_b_*) and the peak correlation coefficient CC_<*q*peak>_(ρ*_a_*, ρ*_b_*), are implemented in *PHENIX* (Adams *et al.*, 2010 ▶) and are also available as an independent program from AU.

## Supplementary Material

Supporting Information.. DOI: 10.1107/S1399004714016289/kw5094sup1.pdf

## Figures and Tables

**Table 1:** Numerical comparison of the syntheses for the peptide model For definition of the syntheses (Fig. 1 ▶) and the correlation coefficients between ρ_*a*_ and ρ_*b*_, see the text.

ρ_*a*_	ρ_*b*_	CC	CC_r_	CC_50_	CC_70_	CC_80_	CC_90_	CC_95_	CC_99_
ρ_pept_*a*_	ρ_pept_*b*_	0.895	0.557	0.597	0.663	0.708	0.485	0.517	0.829
ρ_pept_*a*_	ρ_pept_*c*_	0.596	0.219	0.214	0.360	0.488	0.662	0.740	0.428
ρ_pept_*b*_	ρ_pept_*c*_	0.660	0.273	0.210	0.295	0.337	0.349	0.553	0.553

**Table 2:** Comparison of the Fourier syntheses for selected PDB entries All syntheses were obtained with the calculated structure factors (*F*_calc_, ϕ_calc_). Correlation coefficients between ρ_*a*_ and ρ_*b*_ are defined in the text.

PDB code	ρ_*a*_ resolution (Å)	ρ_*b*_ resolution (Å)	CC	CC_r_	CC_50_	CC_70_	CC_80_	CC_90_	CC_95_	CC_99_
1q09	2–∞	2–10	0.714	0.059	0.080	0.242	0.471	0.811	0.850	0.669
	4–∞	4–10	0.605	0.495	0.353	0.388	0.510	0.680	0.591	0.238
	4–∞	2–∞	0.876	0.441	0.560	0.825	0.847	0.629	0.438	−0.070
1zud	2–∞	2–10	0.855	0.443	0.666	0.864	0.908	0.899	0.875	0.845
	4–∞	4–10	0.764	0.622	0.670	0.764	0.767	0.683	0.590	0.459
	4–∞	2–∞	0.797	0.779	0.685	0.555	0.468	0.260	0.002	−0.402
1ous	2–∞	2–10	0.961	0.860	0.868	0.958	0.962	0.954	0.938	0.908
	4–∞	4–10	0.888	0.832	0.808	0.846	0.857	0.820	0.745	0.588
	4–∞	2–∞	0.596	0.492	0.419	0.331	0.239	0.005	−0.250	−0.528

**Table 3:** Influence of excluded test data sets Fourier syntheses at the resolution *d*_high_ = 2 Å were calculated for the IF2 structure (Simonetti *et al.*, 2013 ▶) using *F*_obs_ or *F*_model_ amplitudes and phases ϕ_model_. Correlation coefficients between ρ_*a*_ and ρ_*b*_ are defined in the text.

Type of amplitudes	ρ_*a*_, data excluded (%)	ρ_*b*_, data excluded (%)	CC	CC_r_	CC_50_	CC_70_	CC_80_	CC_90_	CC_95_	CC_99_
*F*_obs_	0	5	0.976	0.900	0.783	0.805	0.938	0.966	0.957	0.905
		10	0.951	0.842	0.694	0.786	0.887	0.936	0.920	0.834
		20	0.899	0.753	0.591	0.684	0.792	0.869	0.841	0.705
*F*_model_	0	5	0.974	0.850	0.687	0.846	0.952	0.963	0.954	0.895
		10	0.950	0.797	0.627	0.788	0.905	0.935	0.918	0.824
		20	0.900	0.715	0.547	0.691	0.813	0.873	0.840	0.685

**Table 4:** Influence of amplitudes and bulk-solvent modelling The Fourier syntheses were calculated with the complete data sets at resolution *d*_high_ for the IF2 structure (Simonetti *et al.*, 2013 ▶). Correlation coefficients between ρ_*a*_ and ρ_*b*_ are defined in the text.

*d*_high_ (Å)	ρ_*a*_, coefficients	ρ_*b*_, coefficients	CC	CC_r_	CC_50_	CC_70_	CC_80_	CC_90_	CC_95_	CC_99_
2	*F*_obs_, ϕ_model_	*F*_model_, ϕ_model_	0.970	0.834	0.671	0.843	0.940	0.944	0.926	0.819
2	*F*_calc_, ϕ_calc_	*F*_model_, ϕ_model_	0.894	0.623	0.712	0.905	0.940	0.963	0.961	0.953
3	*F*_calc_, ϕ_calc_	*F*_model_, ϕ_model_	0.881	0.684	0.733	0.915	0.949	0.941	0.925	0.864

## Footnotes

1 Supporting information has been deposited in the IUCr electronic archive (Reference: KW5094).

## Appendix A Rank-scaled synthesis and corresponding statistical moments

Firstly, for a synthesis calculated on an arbitrary scale on a grid composed of *N*_grid_ nodes **n**, one computes the frequency of its values, as was introduced into crystallography by Lunin (1988 ▶) and Main (1990*a* ▶,*b* ▶). To do so, the interval (ρ_min_, ρ_max_) = [min ρ(**n**), max ρ(**n**)] is divided into *J* nonintersecting subintervals called bins: (ρ_0_ = ρ_min_, ρ_1_), (ρ_1_, ρ_2_), …, (ρ_*J*−1_, ρ_*J*_ = ρ_max_). For each grid node **n**, we identify the interval *j* to which the corresponding value ρ(**n**) belongs to,

and add a unit to the counter *n_j_* of this bin. The frequencies of the synthesis values are then calculated as

giving

The quantile ranks *q_j_* corresponding to the bin borders μ = ρ*_j_* are the numbers *N*(ρ*_j_*) of grid nodes **n** with the synthesis value below the threshold, ρ(**n**) < ρ*_j_*, normalized as

For an intermediate value ρ_*j*−1_ < μ < ρ*_j_*, its quantile rank *q* may be calculated by a linear interpolation

making it a strictly increasing function of the initial synthesis values. Inversely, for a given rank *q*_*j*−1_ < *q* < *q* *_j_* the corresponding initial synthesis value is recovered as

The value complementary to *q*(μ; ρ) gives the fractional volume *V*_f_ of the unit cell selected by the corresponding cutoff level

Scaling of crystallographic Fourier syntheses in fractional volume has been described previously, for example by Vernoslova & Lunin (1993 ▶).

Let Q(**n**) be a rank-scaled synthesis where each value ρ(**n**) is substituted by the corresponding quantile rank using (28). We split the nodes into *M* equal groups defined by the values of the synthesis, with no relation to their position in the cell. The first *N*_grid_ points correspond to the lowest values of the synthesis; the corresponding rank values are 0 < *Q*(**n**) < 1/*M*; the next *N*_grid_/*M* points correspond to slightly higher values with 1/*M* < *Q*(**n**) < 2/*M* *etc*. Then, for large enough *M*,

Similarly,
