# Supplementary material for: Metrics for comparison of crystallographic maps
Source: Acta Crystallogr D Biol Crystallogr. 2014 Sep 27;70(Pt 10):2593–606. doi: 10.1107/S1399004714016289 (PMC4188004; doi:10.1107/S1399004714016289)
Supplement: Supplementary file 1 [file d-70-02593-sup1.pdf]

METRICS FOR COMPARISON OF CRYSTALLOGRAPHIC MAPS

Alexandre Urzhumtsev[1,2,*], Pavel V. Afonine[3], Vladimir Y. Lunin[4], Thomas C. Terwilliger[5], Paul D. Adams[3,6]

[1]*Centre for Integrative Biology, Department of Integrated Structural Biology, IGMBC, CNRS UMR 7104 – INSERM U964 – Université de Strasbourg, 1 rue Laurent Fries, BP 10142, 67404 Illkirch, France*

[2]*Université de Lorraine, Faculté des Sciences et Technologies, Vandoeuvre-lès-Nancy, 54506, France*

[3]*Lawrence Berkeley National Laboratory, One Cyclotron Road, BLDG 64R0121, Berkeley, CA 94720 USA*

[4]*Institute of Mathematical Problems of Biology, Russian Academy of Sciences, Pushchino, 142290 Russia*

[5]*Los Alamos National Laboratory, Los Alamos, NM 87545-0001*

[6]*Department of Bioengineering, University of California Berkeley, Berkeley, CA 94720 USA*

[*]e-mail: sacha@igbmc.fr

S1. Comparison of two grid functions

Let us have two functions calculated at the same set of points, $\{\mathbf{n}\}$. In particular, they may be two Fourier syntheses $\rho_a(\mathbf{n})$ and $\rho_b(\mathbf{n})$ calculated on the same three-dimensional grid. A difference between these two sets of values may be expressed using as a least-squares target

$$LS(\rho_a,\rho_b) = \sum_{\mathbf{n}} \left[\rho_a(\mathbf{n}) - \rho_b(\mathbf{n})\right]^2 =$$
$$= \left[\sum_{\mathbf{n}} \rho_a^2(\mathbf{n})\right] + \left[\sum_{\mathbf{n}} \rho_b^2(\mathbf{n})\right] - 2\sum_{\mathbf{n}} \rho_a(\mathbf{n})\rho_b(\mathbf{n}) \tag{SA.1}$$

This may be considered as a square distance between two corresponding vectors. To have a measure independent of linear scaling and of the number of points, in macromolecular crystallography metric (A.1) is traditionally replaced by the correlation coefficient

$$CC(\rho_a,\rho_b)=\frac{\sum_{\mathbf{n}}\left(\rho_a(\mathbf{n})-\langle\rho_a\rangle\right)\left(\rho_b(\mathbf{n})-\langle\rho_b\rangle\right)}{\sqrt{\sum_{\mathbf{n}}\left(\rho_a(\mathbf{n})-\langle\rho_a\rangle\right)^2}\sqrt{\sum_{\mathbf{n}}\left(\rho_b(\mathbf{n})-\langle\rho_b\rangle\right)^2}} \qquad \text{(SA.2)}$$

In crystallographic literature, this is addressed for example by Read, 1986; Lunin & Woolfson, 1993; Lunin *et al.*, 2000, etc. When the Fourier syntheses are calculated without the term F_{000}, the mean values for both syntheses are equal to zero

$$\langle\rho_a\rangle=\langle\rho_b\rangle=0 \qquad \text{(SA.3)}$$

reducing (SA.2) to

$$CC(\rho_a,\rho_b)=\frac{\sum_{\mathbf{n}}\rho_a(\mathbf{n})\rho_b(\mathbf{n})}{\sqrt{\sum_{\mathbf{n}}\rho_a^2(\mathbf{n})}\sqrt{\sum_{\mathbf{n}}\rho_b^2(\mathbf{n})}} \qquad \text{(SA.4)}$$

This value may be calculated either directly or through corresponding Fourier coefficients (Read, 1986; Lunin & Woolfson, 1993). When $\sum_{\mathbf{n}}\rho_a^2(\mathbf{n})=\sum_{\mathbf{n}}\rho_b^2(\mathbf{n})$, which is the case when the syntheses are calculated with the same amplitudes whatever the phases are, (SA.1) and (SA.4) are related to each other as

$$\frac{LS(\rho_a,\rho_b)}{2\sum_{\mathbf{n}}\rho_a^2(\mathbf{n})}=1-CC(\rho_a,\rho_b) \qquad \text{(SA.5)}$$

The maximal value of (A.4) is equal to 1 when the syntheses are proportional to each other

$$\rho_b(\mathbf{n})=\lambda\rho_a(\mathbf{n}),\ \ \lambda>0\ , \qquad \text{for all } (\mathbf{n}) \qquad , \qquad \text{(SA.6)}$$

is equal to -1 for anti-proportional syntheses and is equal to 0 if the syntheses are irrelevant (uncorrelated) to each other.

S2. Comparison of artificial grid functions $\tau(\mathbf{n})$

S.2.1. Comparison of grid functions

We use the same peptide model as in main text (Section 3.1) for another example showing that the new metrics can be applied to any grid functions and not only to crystallographic syntheses. Three functions, calculated on the same grid as previously, have been generated analytically to know exactly what happened in each grid node and to illustrate more clearly how the new metrics work.

To construct these grid functions we introduced a theoretical crystal (space group $P1$, unit cell parameters $a = b = 6$ Å, $c = 3$ Å, $\alpha = \beta = \gamma = 90°$) containing an idealized peptide model. For each grid function, the contribution of all 'atoms' was the same being defined by the distance-dependent function $f(r)$ containing a peak in the origin and a negative ripple. Only one atom, the closest one, contributed to each grid node. We introduced first two distance-dependent functions $f_a(r)$ and $f_b(r)$. They were composed from a peak in the origin and a 'ripple'; they differed both in the peak shape and in the position of the 'ripple' (Fig. S1.a) :

$$f_a(r) = \begin{cases} f_{a,inn} = \dfrac{0.01}{0.01 + r^2} - 0.0099 & for \quad r \le 1 \\ f_{a,out} = \exp\left(-16(r - 1.5)^4\right) & for \quad r > 1 \end{cases} \qquad \text{(SB.1)}$$

$$f_b(r) = \begin{cases} f_{b,inn} = \exp\left(-4r^2\right) - 0.0183 & for \quad r \le 1 \\ f_{b,out} = \exp\left(-16(r - 2.5)^4\right) & for \quad r > 1 \end{cases} \qquad \text{(SB.2)}$$

The coefficients in (SB.1) and (SB.2) are adjusted to make the functions continuous.

Then for each grid node **n**, we calculated the distance $r(\mathbf{n})$ to the closest atom of the peptide group taking into account periodicity conditions, and then assigned the function value accordingly to two distance-dependent functions chosen, $\tau_a(\mathbf{n}) = f_a(r(\mathbf{n}))$ or $\tau_b(\mathbf{n}) = f_b(r(\mathbf{n}))$

The conventional map correlation coefficient $CC(\tau_a, \tau_b)$ between the obtained grid functions $\tau_a(\mathbf{n})$ and $\tau_b(\mathbf{n})$ is equal to 0.32. The rank correlation coefficient $CC_r(\tau_a, \tau_b)$ is even lower because the grid points with low function values (negative in the initial scale, before rank scaling) are different, they are at a different distance from the 'atomic centers' (compare f_a

with f_b for $r > 1$, Fig. S1.a), and the corresponding isosurfaces are also different. However the correlation coefficient $CC_{90}(\tau_a, \tau_b)$ with a cutoff of q_{peak} = 0.90 is equal to 1.00 showing that the maps corresponding to the peaks of the functions $\tau_a(\mathbf{n})$ and $\tau_b(\mathbf{n})$ coincide. It is important to note that in the initial scale the peak shape is different for these two functions (Fig. S1.a; Figs. S2.a, b); nevertheless, the maps taken at the same rank values are indeed the same (compare Fig. S2.a with Fig. S2.c). Table S1 gives the value of several other correlation coefficients for these functions.

The curve $D(q; \tau_a, \tau_b)$ = 0 for $q \geq 0.86$ (Fig. S1.b) confirms that the corresponding high-values maps, when taken at the equal percentile, are exactly the same even when the peak shapes for f_a and f_b are very different from each other (Fig. S1.a). The values of $D(q; \tau_a, \tau_b) \approx 1$ or even above it for q < 0.60-0.65 show that at a lower cut-off level the masks are unrelated to each other thus explaining the low $CC(\tau_a, \tau_b)$ and $CC_r(\tau_a, \tau_b)$ values.

We used the same scheme to build one more grid function, $\tau_c(\mathbf{n})$, combining the central peak of f_a with the ripple of f_b as:

$$f_c(r) = \begin{cases} f_{a,inn} = \dfrac{0.01}{0.01 + r^2} - 0.0099 & for \quad r \leq 1 \\ f_{b,out} = \exp\left(-16(r - 2.5)^4\right) & for \quad r > 1 \end{cases} \quad . \qquad \text{(SB.3)}$$

Now $CC_r(\tau_c, \tau_b) \equiv 1$ in spite of a difference in the shape of the peaks. We would not know this peaks coincidence from the conventional correlation $CC(\tau_c, \tau_b)$ = 0.723 which is significantly below 1.0 since the functions' values are not proportional to each other.

Note also that comparison of $\tau_a(\mathbf{n})$ with $\tau_b(\mathbf{n})$ and $\tau_a(\mathbf{n})$ with $\tau_c(\mathbf{n})$ gives the same correlations $CC_{<ppeak>}$ (small differences are due to computational round-off) since $\tau_b(\mathbf{n})$ can be obtained from $\tau_c(\mathbf{n})$ by a monotonic non-linear transformation in the peak region and coincides with $\tau_c(\mathbf{n})$ otherwise.

S3. Map comparison using histogram matching

When calculating CC (equation 4) or CC_r (equation 17) for two grid functions $\rho_a(\mathbf{n})$ and $\rho_b(\mathbf{n})$, we apply the same operation to the same set of grid nodes with the only difference that we compare different functions assigned to these grid nodes – saying more accurately, the same $\rho_a(\mathbf{n})$ and $\rho_b(\mathbf{n})$ but scaled differently. In equation (17) the rescaled functions are $Q_a(\mathbf{n})$ and $Q_b(\mathbf{n})$; such the scaling is also used in digital image processing and called *histogram equalization* (see for example Pratt, 1978). Generally speaking, another scaling would give two other functions $\tau_a(\mathbf{n})$ and $\tau_b(\mathbf{n})$. We require from them that the sets $M_a = \{\mathbf{n} : \tau_a(\mathbf{n}) < \mu\}$ and $M_b = \{\mathbf{n} : \tau_b(\mathbf{n}) < \mu\}$ contain the same number of grid nodes when the same cut-off level μ is applied:

$$\eta(\mu; \tau_a(\mathbf{n})) \equiv \eta(\mu; \tau_b(\mathbf{n})) \qquad \text{(SC.1)}$$

(see equation (10) for definition of $\eta(\mu; \tau(\mathbf{n}))$). Obviously, the substitution of $\rho_a(\mathbf{n})$ and $\rho_b(\mathbf{n})$ by the corresponding quantile ranks, $\tau_a(\mathbf{n}) = \eta(\rho_a(\mathbf{n}); \rho_a)$ and $\tau_b(\mathbf{n}) = \eta(\rho_b(\mathbf{n}); \rho_b)$, is a trivial but not the only way to fulfill the condition (SC.1).

To do this in a general way, we define some reference function $\eta_0(\mu)$ strictly increasing from 0 to 1. Then we modify the syntheses values replacing $\rho_a(\mathbf{n})$ and $\rho_b(\mathbf{n})$ by $\tau_a(\mathbf{n})$ and $\tau_b(\mathbf{n})$, respectively :

$$\rho_a(\mathbf{n}) \to \tau_a(\mathbf{n}) = T_a(\rho_a(\mathbf{n}); \eta_0) \quad , \quad \rho_b(\mathbf{n}) \to \tau_b(\mathbf{n}) = T_b(\rho_b(\mathbf{n}); \eta_0) \qquad \text{(SC.2)}$$

so that for both modified functions their rank values coincide with $\eta_0(\mu)$:

$$\eta(\mu; \tau_a) \equiv \eta(\mu; \tau_b) \equiv \eta_0(\mu) \qquad \text{(SC.3)}$$

The procedure is similar to the crystallographic histogram matching (Zhang & Main, 1990; Lunin & Vernoslova, 1991) and the transformations (SC.2) depend on the value of the synthesis and not on the position of the grid node **n**. When both modifying functions $T_a(\rho)$ and $T_b(\rho)$ are strictly monotonous, the transformation preserves the shape of the isosurfaces

simply changing the associated value. After modification (SC.2), the correlation coefficient between the modified functions $\tau_a(\mathbf{n})$ and $\tau_b(\mathbf{n})$ is calculated:

$$CC_h(\rho_a, \rho_b; \eta_0) = CC(\tau_a, \tau_b) \tag{SC.4}$$

Various options may be suggested to define the reference function $\eta_0(\mu)$. We may choose $\eta_0(\mu)$ equal to the cumulative frequency distribution of one of the syntheses, *e.g.* that of $\rho_b(\mathbf{n})$. This is convenient if we wish to compare several syntheses with a given one, since it makes comparable all syntheses in question and defines a unique scale. In addition, this simplifies the calculations making unnecessary the modification of the second synthesis, $\tau_b(\mathbf{n}) \equiv \rho_b(\mathbf{n})$. An inconvenience of such a choice is that this function is, generally speaking, non commutative:

$$CC_h(\rho_a, \rho_b; \eta(\mu; \rho_b)) \neq CC_h(\rho_b, \rho_a; \eta(\mu; \rho_a)) \tag{SC.5}$$

Alternatively, one may take $\eta_0(\mu)$ equal to some external reference distribution, for example calculated from the predicted histogram (*e.g.*, Lunin & Skovoroda, 1991). Such external reference histograms are used in other programs, for example in *RESOLVE* (Terwilliger, 2000).

As mentioned above, a particular case of an external reference function is

$$\eta_0(\mu) = \eta_{rank}(\mu) \equiv \mu \quad , \qquad 0 \le \mu \le 1 \tag{SC.6}$$

when the synthesis values are replaced by the values of corresponding quantile ranks and for which $CC_h(\rho_a, \rho_b; \eta_{rank}) = CC_r(\rho_a, \rho_b)$. Inversely, calculating (SC.4) with $\eta_0(\mu) \neq \eta_{rank}(\mu)$ may be considered as $CC_r(\rho_a, \rho_b)$ with non uniformly weighted percentile values.

Table S1. Numerical comparison of the artificial grid functions for the peptide model.

		CC	CC_r	CC_{50}	CC_{70}	CC_{80}	CC_{90}	CC_{95}	CC_{99}
τ_a	τ_b	0.321	-0.282	-0.056	0.310	0.827	1.000	1.000	1.000
τ_b	τ_c	0.723	1.000	1.000	1.000	1.000	1.000	1.000	1.000
τ_a	τ_c	-0.230	-0.284	-0.062	0.302	0.821	1.000	1.000	1.000

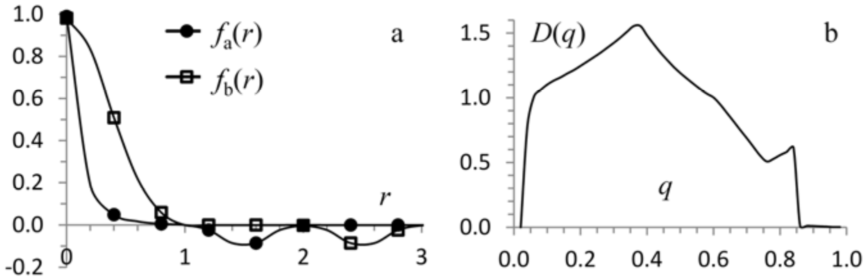

Fig. S1. Comparison of the peptide 'mathematical' functions. a) Distance-dependent functions $f_a(r)$ (solid circular markers) and $f_b(r)$ (open squares markers) used to define the grid functions $\tau_a(\mathbf{n})$, $\tau_b(\mathbf{n})$ and $\tau_c(\mathbf{n})$. **b)** Discrepancy function $D(q)$ between $\tau_a(\mathbf{n})$ and $\tau_b(\mathbf{n})$. $D(q)$ values equal to 0 for q > 0.86 indicate a full coincidence of the peaks when the functions are rank scaled.

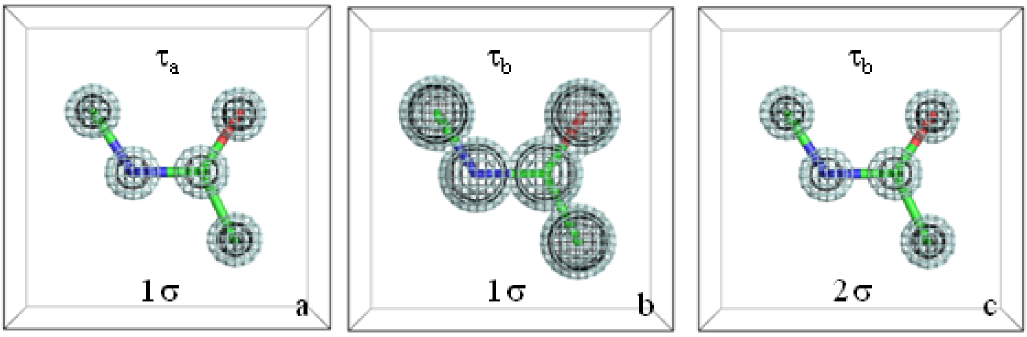

Fig. S2. Maps corresponding to functions $\tau_a(\mathbf{n})$ and $\tau_b(\mathbf{n})$. **a)** Function $\tau_a(\mathbf{n})$; cut-off levels 1 σ (light blue) and 2 σ (dark blue). **b)** Function $\tau_b(\mathbf{n})$; the same cut-off levels in σ as for (a). **c)** Function $\tau_b(\mathbf{n})$; cut-off levels 2.7 σ (light blue) and 5.3 σ (dark blue) corresponding to the same rank levels as in (a).
